# Processing stimulus dynamics by the NF-κB network in single cells

**DOI:** 10.1038/s12276-023-01133-7

**Published:** 2023-12-01

**Authors:** Minjun Son, Andrew G. Wang, Bijentimala Keisham, Savaş Tay

**Affiliations:** 1 Pritzker School of Molecular Engineering, University of Chicago, Chicago, IL 60637 USA; 2 Institute for Genomics and Systems Biology, University of Chicago, Chicago, IL 60637 USA; 3 Medical Scientist Training Program, University of Chicago, Chicago, IL 60637 USA

**Keywords:** Extracellular signalling molecules, NF-kappaB

## Abstract

Cells at the site of an infection experience numerous biochemical signals that vary in amplitude, space, and time. Despite the diversity of dynamic signals produced by pathogens and sentinel cells, information-processing pathways converge on a limited number of central signaling nodes to ultimately control cellular responses. In particular, the NF-κB pathway responds to dozens of signals from pathogens and self, and plays a vital role in processing proinflammatory inputs. Studies addressing the influence of stimulus dynamics on NF-κB signaling are rare due to technical limitations with live-cell measurements. However, recent advances in microfluidics, automation, and image analysis have enabled investigations that yield high temporal resolution at the single-cell level. Here, we summarize the recent research which measures and models the NF-κB response to pulsatile and fluctuating stimulus concentrations, as well as different combinations and sequences of signaling molecules. Collectively, these studies show that the NF-κB network integrates external inflammatory signals and translates these into downstream transcriptional responses.

## Decoding infection: NF-κB pathway’s vital role in processing signal dynamics

Minjun Son’s research focuses on the intricate regulation of NF-κB, a central transcription factor governing inflammatory responses, under diverse stimulus dynamics and combinations. These stimuli include cytokines (small secreted proteins for cell-cell signaling) and other secretions from pathogens. While the precise mechanisms remain unknown, previous and ongoing studies have endeavored to elucidate NF-κB’s response to short bursts, fluctuations, or combinations of stimuli. Through advanced technologies and analytical methods, researchers discovered that the NF-κB response is significantly influenced by the dynamics and combinations of stimuli. This variance in NF-κB response resulted in distinct gene expression patterns, underscoring the pivotal role of the NF-κB pathway in interpreting the dynamics of signal molecules and determining cell behavior. Future studies employing mouse models would bridge the gap between these experimental findings and physiological processes in vivo.

This summary was initially drafted using artificial intelligence, then revised and fact-checked by the author.

## Introduction

Immune cells sense and respond to a wide range of signaling molecules from pathogens and host tissue through a limited number of signaling pathways. These pathways must integrate information about the dose, dynamics, and combinations of environmental stimuli to coordinate appropriate immune responses. NF-κB family transcription factors play key roles in translating extracellular signals into cellular responses. NF-κB activation is regulated by dozens of signaling molecules, including cytokines, such as tumor necrosis factor (TNF-α), interleukin 1 beta (IL-1β), and interleukin 17 (IL-17), and pathogenic molecules, such as lipopolysaccharides (LPS), lipopeptides, and double-stranded RNA^1–3^. Each signal activates a particular receptor that recruits various intracellular proteins in a ubiquitination and kinase cascade that triggers the activation and nuclear translocation of NF-κB^1,4^. In the nucleus, NF-κB promotes the transcription of genes involved in a number of cellular processes, including inflammation, cell survival, growth, differentiation, and apoptosis^3,5–7^. NF-κB therefore plays a critical role in integrating and interpreting inflammatory signals. Polymorphisms of components in the NF-κB pathway and dysregulated NF-κB nuclear translocation have been associated with autoimmune diseases such as rheumatoid arthritis, inflammatory bowel disease, and psoriasis^8,9^, and knocking out NF-κB was shown to be embryonically lethal^10^. NF-κB signaling is particularly notable due to the importance of activation dynamics in the regulation of downstream transcriptional activity. Different inflammatory stimuli produce distinct patterns of activation dynamics, including both oscillatory and pulsatile response phenotypes^11–15^. These stimulus-specific activation dynamics are then translated into distinct transcriptional and epigenetic phenotypes^14,16,17^.

Inflammation-associated signaling is highly dynamic in vivo. Both systemic models of inflammation and ex vivo studies of tissue-specific inflammatory responses reveal different cytokine dynamics in response to pathogenic challenge. Endotoxemia in mouse models resulted in an exponential increase in plasma TNF-α and IL-6 levels for up to 20 h^18^. Similarly, ex vivo human lung tissue treated with LPS rapidly produced TNF-α and IL-1β for several hours, followed by production of IL-6, IL-8, and IL-10 in the later phase^19^. Transient endotoxemia in healthy adults increased the plasma level of TNF-α, an early response cytokine, for the first 2–3 h after bolus infusion and before gradually decreasing^20^. Furthermore, following the increase of TNF-α, the levels of IL-1β, IL-6, and IL-10 increased sequentially, showing different dynamics and sequences of cytokine production in the human response to immune challenge. Other ex vivo studies have also highlighted the complex profiles of cytokine secretion by primary monocytes or macrophages^21–24^. In particular, Tassi et al. demonstrated that the kinetics of IL-1β secretion varied widely, from a slow linear increase to a rapid exponential increase, depending on pathogenic stimulus, suggesting stimulus-specific secretion dynamics induced by sentinel cells^22^. Human monocytes and macrophages in a population secreted TNF-α, IL-1β, IL-6, IL-8, and IL-10 at different dynamics compared to those cells in an isolated environment, indicating that paracrine signaling changes cytokine secretion dynamics^23^. To overcome infection, cells must respond to these dynamic changes in environmental cytokine levels and adjust their behaviors accordingly. As a central part of the process by which cells respond to an inflammatory microenvironment, the NF-κB network plays a key role in sensing these dynamics and producing appropriate transcriptional responses. Interestingly, a number of these cytokines are both activators of NF-κB signaling and produced by activated NF-κB. As a result, there is a potential that the initial signal dynamics not only influence the NF-κB activation dynamics, but also impact the subsequent cytokine levels and downstream outcome through positive feedback. However, despite the importance of cytokine dynamics in the immune response, studies addressing the effect of cytokine dynamics are still rare.

Investigating how signaling networks interpret stimulus dynamics may reveal previously unknown aspects of the signaling network. For example, profiling transcription factor activity under different input dynamics has led to the identification of key network components as targets of pharmaceutical perturbation^25^. Pulsatile stimulation mediated by inflammatory ligands has revealed how stochastic chemical reactions in the signaling network increase NF-κB activation and subsequent downstream gene expression^17^. However, despite the importance of these works on dynamical inputs to the NF-κB network, comprehensive studies that systematically investigate the impact of input dynamics on NF-κB response are still uncommon. This scarcity is partially due to challenges in comprehensively testing different input dynamics in high-throughput to effectively characterize the rules governing input-to-output processing, which requires continuous and precise modulation of stimulus dynamics in multiple samples while monitoring cell responses in real time.

Here, we review early and recent advances in testing the effect of various stimulus dynamics, sequences, and combinations on the NF-κB network. We first provide an overview of the NF-κB signaling pathway, compare the results from various stimulation patterns, and then discuss how NF-κB activity effectively encodes stimulus dynamics and the implications of this process for an overall immune response.

## Overview of the NF-κB signaling network

**Fig. 1:**
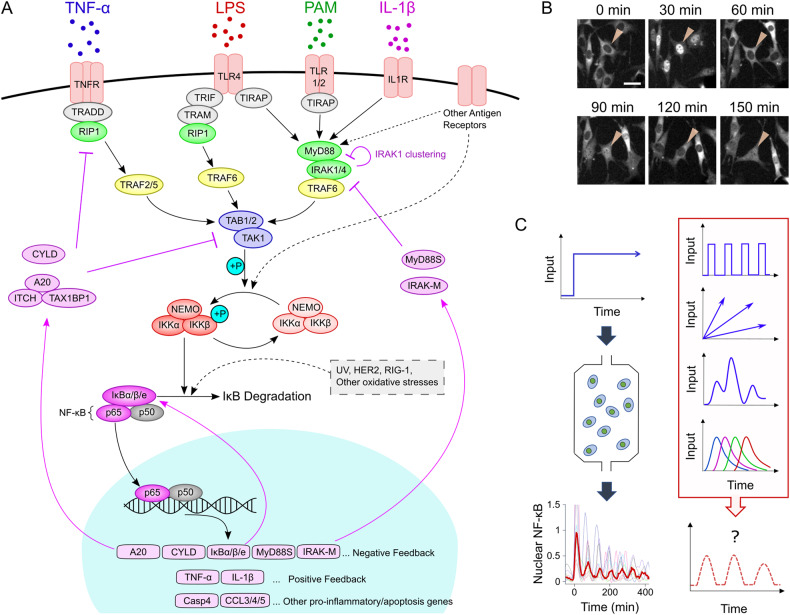
The canonical NF-κB pathway and its oscillatory dynamics. **A** Pathway diagram illustrating NF-κB activation through initiation of nuclear translocation by various extracellular signaling molecules. The purple arrows indicate the expression of various negative feedback proteins targeting different intermediary proteins or kinases in the upstream pathway. **B** Time-lapse fluorescence images show the oscillation of p65-RFP nuclear localization in fibroblasts. **C** Images in the left column show the damping NF-κB oscillation in response to continuous TNF-α stimulation, while images in the right column show possible stimulus dynamics in vivo. The input pattern in the fourth row of the right column illustrates different ligands accumulating and degrading in a different order. The images in (**B**, **C**) are adapted from Son et al. with permission^13^.

The NF-κB family of transcription factors includes homo-and heterodimers composed of RelA (p65), RelB, c-Rel, p50, and p52 subunits^26^. The p65/p50 heterodimer is the most abundant dimer in almost all cell types^27^. Prior to stimulation, NF-κB dimers are bound to a series of inhibitor proteins, named inhibitor of kappa B (IκB) protein isoforms, and the nuclear translocation and transcription activity of these dimers are generally blocked by IκB proteins. IκB can be further classified into four known isoforms, IκBα, IκBβ, IκBε, and IκBγ. Each IκB isoform is degraded at different rates upon induction, and shows different affinity for Rel proteins^28–33^. p100, which has also been termed “IκBδ”, is the unprocessed form of p52 that acts like an IκB protein and blocks NF-κB dimers from nuclear translocation^34^. We focus on the canonical p65/p50 and p65/p52 heterodimers and summarize the signaling pathway associated with them (Fig. 1A).

### The TNFα – NF-κB signaling module

TNF-α is a central inflammatory cytokine primarily secreted by macrophages, T cells, and natural killer cells in response to pathogenic stimuli^8,35,36^. Antibodies neutralizing TNF-α activity are among the most successful drugs for treating chronic inflammation and other autoimmune diseases^36^. TNF-α predominantly exists as a homotrimer, which binds and activates the homotrimeric form of its receptor, TNFR^37–40^. Activated TNFR recruits intermediary proteins such as TRADD, cIAP1/2, RIP1, and TRAF2/5, which then undergo ubiquitination and further recruit the downstream kinases TAB1/2-TAK1 and IKK complexes^8,41–43^. The TAB1/2-TAK1 complex phosphorylates the canonical IKK kinase complex, which consists of two kinases (IKKα and IKKβ) and a regulatory subunit, NEMO^44^. The activated IKK complex facilitates the ubiquitination-mediated degradation of IκB family proteins via phosphorylation, and consequently releases NF-κB, which can be translocated to the nucleus. Nuclear NF-κB binds to a κB motif in the genome and induces the transcription of response genes. These target genes include proinflammatory, proliferative, and apoptotic genes, as well as genes involved in positive and negative regulation of NF-κB signaling, such as pro- and anti-inflammatory cytokines, IκB proteins, and A20^33^. Newly translated IκB binds to free NF-κB in both the nucleus and cytosol, driving the export of NF-κB from the nucleus and sequestering it in the cytosol, resulting in the rapid inhibition of NF-κB activity^45–47^. However, if it remains activated, the IKK complex facilitates the degradation of newly synthesized IκB proteins and initiates another cycle of NF-κB translocation (Fig. 1B). These repeating cycles, or oscillations, of NF-κB translocation have been the focus of intense research in the past two decades^48–50^. Other negative feedback proteins, such as A20 and CYLD, act upstream of IKK. Both A20 and CYLD are actively expressed by nuclear NF-κB, and inhibit the IKK activation by editing (A20) or deubiquitinating (CYLD) the scaffolds to which IKK binds^51–53^. Additionally, A20 can facilitate the degradation of early members of the signaling cascade to further inhibit signaling^54^. Notably, A20 or IκBα knockout mice died prematurely within 10 days of age, while TNF-α-deficient mice often lived longer than wild-type mice after infusion with a lethal dose of a pathogenic stimulus^55–57^. This highlights the crucial importance of negative feedback proteins in maintaining the proper functioning of the immune system and overall organism viability.

### NF-κB activation by other signaling molecules

In addition to TNF-α, other classical NF-κB activating ligands include pathogen-associated molecular patterns (PAMPS), which bind different members of the Toll-like receptor (TLR) family and include lipopolysaccharide (LPS), bacterial and synthetic lipopeptides such as PAM2CSK4 (PAM), bacterial flagellin, microbial DNA, and double-stranded RNA-like synthetic polyI:C. LPS, a component of the cell wall of gram-negative bacteria and an extensively studied TLR ligand, binds to the homodimeric form of TLR4 in cooperation with cofactors such as CD14 and LPS-binding protein^1,58^. Although there are some key differences, TLR4 signaling largely resembles TNF-α signaling, and these TLR4 and TNF-α pathways share many common components (Fig. 1A). The primary differences are intermediary proteins that activate TAB1/2-TAK1 and IKK. Instead of TRADD, RIP1, and TRAF2/5 in the TNF-α pathway, TLR4 on cell membrane recruits the ‘myddosome’, which is composed of MyD88, IRAK1/4, and TRAF6 (Fig. 1A)^4,43^. Additionally, TLR4 can signal through a MyD88-distinct pathway via endocytosis of TLR4, which activates TRIF-TRAM pathway^58–60^. The signaling pathways involving other TLRs (TLR1/2 and 2/6) for lipopeptides and the pathway for IL-1β include the MyD88, IRAK1/4, and TRAF6 myddosome, similar to the pathway downstream of TLR4^43,61^. Signaling through most other TLRs (TLR5, 7/8, and 9) is also mediated by this myddosome, while TLR3 signaling in response to double-stranded RNA is mediated by the TRIF-TRAM pathway.

Previous studies have revealed that nuclear NF-κB leads to the expression of additional negative feedback proteins, such as IRAK-M and MyD88S which specifically target signal transduction downstream of TLRs and IL-1R, as well as A20 which inhibits the activity of these receptors through the modification of ubiquitin scaffolds on TRAF6^4,62,63^. Additionally, IRAK1 auto-phosphorylation via strong IL-1β and LPS stimulation facilitates its own aggregation and clustering, inhibiting the activation of downstream components in signaling pathway (Fig. 1A)^64^.

In the following sections, we discuss how the complex network of interactions in the NF-κB pathway processes the order and kinetics of input signals and alters the oscillatory dynamics of NF-κB translocation (Fig. 1C).

## NF-κB response to pulsatile stimuli in single cells

Fluorescently tagging NF-κB enabled real-time monitoring of NF-κB translocation at the single-cell level with minimal changes to protein function^65,66^. Since then, many studies have leveraged this reporter to study NF-κB responses to a brief cytokine pulse or a series of pulses. These stimulation patterns are analogous to a burst release of cytokines due to inflammatory cell death, such as pyroptosis or necroptosis^67–69^. These simple experiments provided valuable insight into the regulation of the NF-κB network. For example, an early study using transfected neuroblastoma cells compared NF-κB dynamics after a 5 min pulse of TNF-α and after continuous TNF-α stimulation^70^. They found that continuous TNF-α stimulation was necessary to maintain IKK activation and produce the continuous oscillation of NF-κB translocation and downstream gene expression. Thus, although the oscillatory dynamics of NF-κB translocation partially stems from delayed expression of the inhibitory protein IκB, it still requires input from environmental stimulus. By comparing the results from pulsatile and continuous stimulation with different doses of TNF-α, the same group demonstrated that NF-κB activation mediated by physiologically relevant doses of TNF-α also required the continuous presence of TNF-α^71^.

**Fig. 2:**
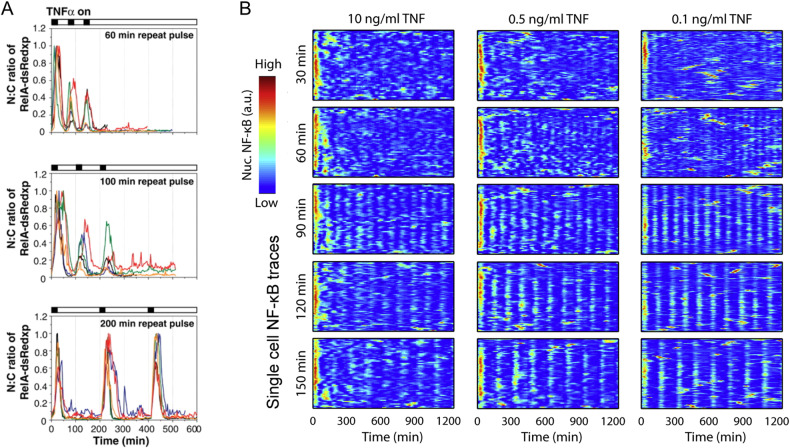
NF-κB response to pulsatile or saw-tooth TNF-α stimulation. **A** Neuroblastoma cells were exposed to TNF-α for 15 min every 60, 100, and 200 min (pulsatile stimulation). Each colored line shows a single cell NF-κB response. **B** Using a microfluidic device, fibroblasts were stimulated with various doses of TNF-α every 30, 60, 90, 120, and 150 min. Due to degradation and internalization of TNF-α by cells, this created sawtooth-like TNF-α kinetics inside cell chambers. The heatmaps show the single-cell NF-κB traces for each condition. The figures are reproduced from Ashall et al. and Kellogg and Tay with permission^17,72^.

Pulsatile TNF-α stimulation over different frequencies revealed additional novel characteristics of the NF-κB system. When TNF-α pulses were introduced at low frequency ( ~ 200 min period between pulses) to neuroblastoma cells, full amplitude NF-κB responses continued even after 8 h^72^. However, at higher frequencies (<100 min period), the NF-κB response amplitude diminished after each pulse, providing insight into the time scale necessary to reset the NF-κB system (Fig. 2A). Interestingly, a preceding mathematical model that accurately described NF-κB dynamics under continuous stimulation failed to reproduce these results with different pulsatile frequencies. This observation led directly to the key finding that IKK cycling between active and inactive states is necessary for NF-κB signal transduction (Fig. 1A)^25^. Another study using high-frequency TNF-α pulses revealed that cells do not respond to restimulation shortly after NF-κB leaves the nucleus (~60 min)^73^. This group demonstrated that this refractory state during NF-κB oscillation was established upstream of IKK activation, and largely depended on the negative feedback protein A20 (Fig. 1A), which highlights the different roles and timeframes of upstream negative feedback modules.

The introduction of microfluidic devices has improved the throughput and precision of pulsatile experiments by increasing the number of simultaneously testable conditions and increasing temporal control over stimulus introduction. Pulsatile microfluidic experiments have revealed the role of stochastic chemical interactions in NF-κB oscillations and the effect of NF-κB entrainment on downstream gene expression^74–76^. Two studies utilized a high-throughput microfluidic device to repeatedly supply different doses of TNF-α to fibroblasts at varying frequencies^17,77^. Due to the degradation and internalization of TNF-α by cells, this feeding pattern resulted in oscillatory, sawtooth-shaped TNF-α dynamics. When the feeding frequency matched the NF-κB oscillation frequency ( ~ 90 min), single-cell behaviors became synchronized, and NF-κB oscillation became more robust, continuously exhibiting high amplitudes in subsequent peaks (Fig. 2B). This work also highlighted how noise in NF-κB oscillation facilitates entrainment and downstream gene expression over a wide range of feeding frequencies^17^. Interestingly, noise also enabled NF-κB oscillations to hop between different entrainment frequencies^77^. Another study supplied pulsatile TNF-α to fibroblasts at different frequencies and doses^78^. They demonstrated that NF-κB oscillation can be synchronized over a broader range of frequencies under pulsatile stimulation patterns than under sawtooth stimulation patterns. This synchronization resulted in different NF-κB dynamics (for example, different oscillatory periods and damping) depending on the frequency and dose of pulses. Interestingly, this group found that although NF-κB oscillation was synchronized, this oscillatory behavior lacked aspects of entrainment, such as an optimal period, which suggests that NF-κB oscillation under repeated stimulation may vary depending on whether the repeated stimulation was applied in a sawtooth or pulsatile pattern. These findings show how different fluctuations of TNF-α in the cellular environment can greatly influence NF-κB oscillation and the resultant inflammatory responses.

Another variation of pulsatile stimulation involves modifying the length and concentration of the pulse. Using a custom-designed microfluidic device, one study varied the duration of TNF-α in a human cell line (HeLa) and monitored NF-κB and caspase-8 signaling^75^. Although a short pulse (<1 min) was enough to activate both pathways, shorter pulse was more likely to induce apoptotic cell death than a longer (~1 h) pulse. Few studies utilize stimuli other than TNF-α for pulsatile experiments. However, one study team varied the amplitude and duration of the LPS pulses, and found that activation probability and oscillatory dynamics correspond to the amplitude and duration of the pulse, respectively^79^.

These findings effectively highlight the utility of pulsatile stimulation in investigating the hidden properties and roles of diverse protein interactions within an intracellular signaling network. This approach also offers insight into how the dynamics of signaling networks influence downstream activities. In the context of NF-κB pathway, a comparison between continuous and varied pulsatile stimulation with TNF-α revealed the pivotal role of subsequent stimulation in sustaining NF-κB oscillation, the contribution of intermediary kinases to NF-κB oscillation, and the involvement of various negative feedback proteins in regulating oscillatory behaviors. Furthermore, downstream analyses revealed a correlation between the expression of NF-κB target genes and the entrainment or synchronization of NF-κB dynamics, which suggests that stable NF-κB oscillation at fixed period can increase downstream gene expression^17,78^.

We also highlight that the characteristics of NF-κB oscillation, such as oscillation period, peak amplitudes, and bandwidth of the peak, significantly vary depending on the cell and stimulus type. For instance, continuous TNF-α stimulation triggered distinct NF-κB oscillations in macrophages, fibroblasts, and epithelial cells^11,12,14,80^, while LPS induced singular activation without exhibiting clear signs of oscillation in these cells^11,14,79^. This difference in the NF-κB response may arise from stimulus-specific negative feedback regulation^81^. Moreover, fibroblasts and epithelial cells exhibited a limited response to polyI:C, possibly due to the weak expression of its receptor (TLR3) and intermediary protein (TRIF), while macrophages showed oscillatory responses^14,15,80^. These results underscore the context-dependent nature of NF-κB response dynamics, which may contribute to the variability in immune gene expression across different cell and stimulus types^80–82^. Experimenting with pulsatile stimulation with various stimuli and cell types can offer mechanistic insights into these disparities. This approach also holds the potential to reveal additional hidden attributes, including variations in the half-life or relaxation time of intermediary proteins, as well as the timing of negative feedback protein activation^71–73^.

## NF-κB response to increasing stimulus dose

**Fig. 3:**
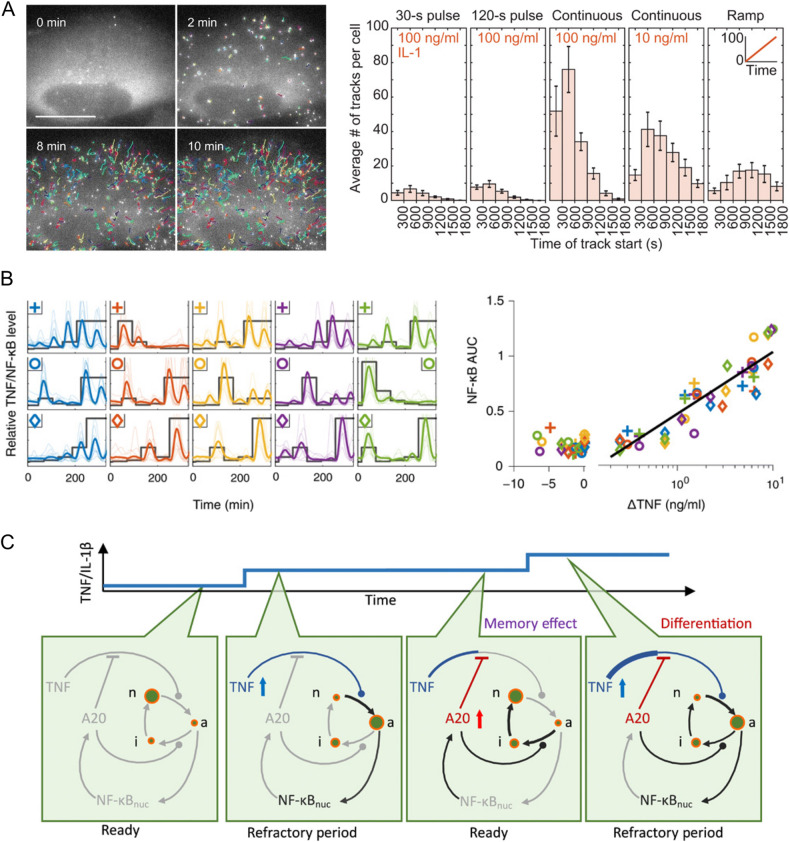
IKK and NF-κB respond to increasing and randomly fluctuating stimuli. **A** U2OS cells expressing NEMO-GFP fusion were stimulated with 100 ng/ml IL-1β. NEMO is a subunit in the IKK complex, and after IKK activation, it is recruited to punctate structures near the cell membrane. On the left, time-lapse images show rapid recruitment of NEMO-GFP toward the cell membrane (colored tracks). On the right, histograms show the number of tracks or recruited NEMO molecules during various stimulus dynamics: pulse, continuous, and linear ramping. **B** Mouse fibroblasts were exposed to randomly fluctuating doses of TNF-α (between 0 and 10 ng/ml). On the left, each subplot shows the different fluctuations (thick gray line in each subplot) and the corresponding NF-κB responses. For each increase or decrease in TNF-α level (ΔTNF) in each sample, the subsequent NF-κB response was quantified and plotted on the right. When combined, the resulting scatter plot shows that the NF-κB response corresponded to positive change in TNF-α level. **C** The diagrams illustrate the core mechanism of the differentiator behavior shown in (**B**). Briefly, TNF-α/IL-1β stimulation caused the initial activation of NF-κB translocation, which subsequently increased the level of the negative feedback protein, A20. However, if the cytokine levels are further increased in the next cycle, it overcomes the inhibition by A20 and initiates the next translocation cycle. Green circles show IKK cycling from neutral (n) to active (a) to inactive (i). The figures are reproduced from Cruz et al. and Son et al. with permission^13,85^.

While the local tissue surrounding sentinel immune cells may be affected by pulse-like stimulation in tissue environment, linearly or exponentially increasing levels of stimuli are more commonly found in plasma and at the systemic level^18–20,22^. However, testing different rates of increasing stimuli is challenging, as it requires generating a wide range of doses and continuous changes in the cellular environment. Although still in pulsatile form of stimulation, one of the earliest studies applied increasing doses of TNF-α pulses to various cancer cell lines to investigate the effects of increasing stimuli^83^. This study revealed that individual cells have different thresholds for NF-κB activation, but once activated, the NF-κB responses increased with increasing doses of TNF-α. To generate gradually increasing input dynamics without pulsing, Mokashi et al. built a microfluidic system that mixed two different media at different ratios and tested the effect of linearly increasing TNF-α on HeLa cells^84^. Although precise and continuous control over the sample environment limited the number of testable conditions, this study highlighted a noteworthy distinction in NF-κB dynamics when cells were subjected to linearly increasing levels of TNF-α compared to those subjected to an instantaneous increase. This result was further described in a recent study by the same group, where they applied different durations of TNF-α and IL-1β pulses and linear increases of both cytokines using the aforementioned mixing device^85^. They compared the kinetics of IKK complex assembly to NF-κB dynamics in individual cells (Fig. 3A) and revealed how the intermediary proteins upstream of IKK encode input dynamics independent of downstream negative feedback regulation (Fig. 1A). Together, these studies demonstrated how applying different stimulus dynamics can reveal hidden characteristics of network regulation.

Although previous studies demonstrated that the NF-κB pathway differentiates between linearly increasing and pulsatile stimuli, how NF-κB in general responds to increasing or fluctuating stimuli occurring in vivo is still unclear. A recent study tried to shed light on this by testing different stimulus dynamics with a high-throughput microfluidic platform, which enabled simultaneously testing in 64 experimental conditions^13^. To test more stimulus dynamics in each experiment, they employed a strategy involving multiple small increases or decreases in TNF-α, IL-1β, and PAM levels administered to mouse fibroblasts, as opposed to the continuous modulation demonstrated in previous examples. Despite subjecting cells to stepwise adjustments using this approach, the team effectively captured the overall NF-κB response to diverse stimulus dynamics and highlighted the significance of stimulus dynamics on the NF-κB response. When they generated linearly or exponentially increasing or randomly fluctuating stimulus patterns, interestingly, they found that only the change in TNF-α and IL-1β levels was reflected in subsequent NF-κB responses, which was analogous to the effect of a differentiator in electronic circuit (Fig. 3B, C). However, increasing the level of PAM, a synthetic analog of bacterial lipopeptide, did not result in differentiator-like behavior. These results highlight that the NF-κB response corresponds to the dynamics of local cytokines, not to absolute doses and that the dynamics of different stimuli may be processed selectively^13^. Notably, in a recent study from the same group focusing on the spatial aspects of NF-κB signaling, they suggested that this differentiator behavior can enable the NF-κB network to interpret detailed information about signal-sending sources, such as the relative distance to local sentinel cells and the levels of cytokine secretion from those cells^76,86^.

Collectively, these findings underscore the multifaceted nature of the translocation dynamics of NF-κB, which encodes information derived from the intricate and evolving signaling milieu during infection. Notably, the oscillatory patterns of NF-κB dynamics exhibit significant divergence based on the dynamics of the applied stimuli, which also contributed to the variations in downstream gene expression. Similar to the observations in experiments with pulsatile stimulation, NF-κB interpretation of stimulus dynamics also varied by cell and stimulus type. Furthermore, the costimulation of cells with multiple stimuli elicited distinct NF-κB responses, depending on the dose and combination of these stimuli^87,88^. These complex responses, arising from diverse stimulus dynamics and combinations, suggest that NF-κB responses are sensitive to the dynamic attributes of environmental stimuli and may give rise to substantially different inflammatory responses depending on stimulus dynamics. Given the prevalence of gradual fluctuations in ligand levels within tissues and plasma^18–24^, these findings underscore the limitations of relying solely on single time-point measurements to gauge the state of the inflammatory response. Thus, the findings presented in these studies emphasize the pivotal role of capturing in vivo ligand dynamics in the study of inflammatory processes.

## NF-κB response to different stimulus sequences and combinations

**Fig. 4:**
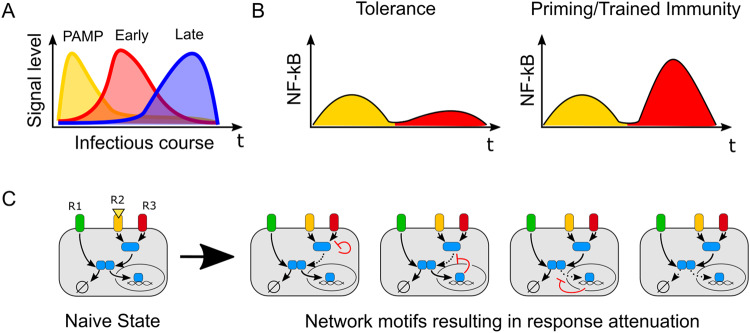
Physiological importance and effects of stimulus sequence and combination on the NF-κB response. **A** Conceptual model of changes in environmental signals over the course of challenge with infection. Pathogenic stimulus (yellow) results in the production of early cytokines (red), followed by late cytokines (blue). **B** Priming and tolerance as two models of how initial inflammatory stimuli can influence subsequent responses to different stimuli. The first stimulus (yellow) can induce either tolerance or priming in response to a subsequent stimulus (red). **C** Multiple examples of network remodeling that exerts distinct effects on signal transduction after exposure to subsequent stimuli. R1, R2, and R3 show three different receptors where R2 and R3 share early signaling intermediaries, while R1 does not share any intermediaries until IKK module. Figure 4c is adapted from Wang et al. with permission^15^.

During an infectious or inflammatory challenge, sentinel cells experience not only the dynamics of a single ligand, but also the complex combinations and sequences of dynamically fluctuating signals (Fig. 4A). For example, in acute endotoxin-induced models of systemic inflammation, serum levels of TNF-α increased within 1 h, while IL-6, IL-10, and IL-1β levels began to rise at 2 h and peaked three to 4 h later^20^. Similarly, in plasma from patients with cytokine storm or septic shock, distinct patterns of pro- and anti-inflammatory cytokine release have been observed and shown to have predictive value for prognosis^89,90^. These paracrine signals play a significant role in coordinating population-level responses when cells are exposed to bacterial or viral stimuli^11,91,92^. Thus, both systemic and local inflammatory responses rely on coordinated and dynamic production of cytokine signals in response to environmental stimuli, which are interpreted by tissue-resident cells in a context-dependent manner.

Several studies have reported that the stimulus sequence and combination can adjust or ‘remodel’ a signaling network through various feedback mechanisms to control subsequent responses^93–95^. In particular, many studies have extensively focused on how innate immune memory is affected by sequential stimulation with repeated or distinct ligands^96–100^. These studies revealed that, over the span of days to weeks, exposure to prior stimuli can attenuate subsequent responses to prevent immune-mediated injury (tolerance) or can potentiate subsequent responses to facilitate pathogen clearance by priming or training immunity (Fig. 4B)^96,101^. Mechanistically, these different responses to sequential stimuli can be achieved through remodeling of chromatin^102–105^ and/or signaling networks^106,107^. However, in many of the studies on stimulation sequences, only timescales on the order of days to weeks were considered, which is appropriate for modeling reinfection or chronic inflammation. Few studies have investigated how rapid network remodeling due to prior stimulus plays a role in the cellular interpretation of evolving inflammatory microenvironments over the course of an infection.

Although many inflammatory stimuli converge on the activation of NF-κB, each activates NF-κB through distinct nodes and controls the response in different ways (Figs. 1A and 4C). For example, many pathogenic TLR ligands, such as LPS and PAM, and acute response cytokines, such as TNF-α and IL-1β, facilitate signaling through NF-κB^43^, while late phase signaling molecules, such as IL-10, sTNFR1, and TGF-β, inhibit signaling mediated through NF-κB^108–110^. Thus, studying the dynamics of NF-κB activation provides a real-time readout of how sequential and combinatorial ligand dynamics are encoded in signaling networks.

In primary mouse embryonic fibroblasts, 24 h of exposure to LPS, TNF-α, or IL-1β resulted in attenuation of the subsequent NF-κB response to LPS, TNF-α, and IL-1β, indicating that the primary effect of sequential stimulation is tolerance on this time scale^31^. Genetic deletion of IκBδ, a noncanonical inhibitor of RelA composed of homodimeric p100, eliminated this tolerance effect, showing that the induction of NF-κB negative feedback is critical to encoding tolerogenic memory (Fig. 1A)^31^. At shorter timescales, when two pulses of TNF-α were introduced to human neuroblastoma cells within 100 min, the second pulse of TNF-α produced a minimal NF-κB response, revealing a refractory period after the first stimulation^73^. However, when the second stimulus was changed to IL-1β, NF-κB was reactivated without any sign of attenuation. These results suggest that although the NF-κB network induces negative feedback that inhibits signal transduction from repeated stimulation with the same ligand, the network can be activated again when it is stimulated with alternative ligands.

Similarly, in mouse fibroblasts, a high dose of IL-1β and LPS shifted cells into a cross-tolerogenic state after 3 h, rendering them insensitive to restimulation with either IL-1β or LPS^64^. However, restimulation with TNF-α after IL-1β or LPS induced weak but noticeable NF-κB activation. The authors identified autophosphorylation and aggregation of the signaling intermediary IRAK1, which is shared by IL-1β and LPS but not by TNF-α, as the critical mechanism of this memory effect (Fig. 1A)^64^. The dose-dependent nature of IRAK1 suggests that IRAK1 autoinhibition can be considered a safety mechanism against excessive inflammation. Moving beyond pairwise stimulation, a recent study stimulated mouse fibroblasts with sequences of four distinct stimuli (TNFα, IL-1β, LPS, and PAM2CSK4) over 8 h and showed that NF-κB dynamics encoded distinct information about the stimulus sequence and identity^15^. Distinct feedback mechanisms encoded memory in the NF-κB signaling network in a dose-dependent and ligand-specific manner to produce distinct NF-κB response patterns when combined. These two studies demonstrated that although sequential stimulation primarily produces tolerance in the NF-κB network, NF-κB response dynamics can be fine-tuned through remodeling of the signaling pathway based on stimulus sequences (Fig. 4C). In addition to tolerance, whether priming or training effects can also take place at the level of transcription factor dynamics is still an open question.

Although few studies have focused on the effect of combinatorial stimuli on NF-κB activation dynamics, Kellogg et al. have shown that when mouse fibroblasts were exposed to both LPS and PAM2CSK4 simultaneously, the resulting NF-κB dynamics predominantly followed the response dynamics by one of the two stimuli, but not both, at the single-cell level^88^. This result raises the possibility that integrative signaling may potentially arise at the population level from individual cells with discrete signaling decisions. In contrast, combining stimuli with non-NF-κB activating stimuli such as Type I and II IFN augmented the NF-κB response from dsRNA (polyI:C) in mouse fibroblasts^87^. These cytokines directly altered the translation and degradation rates of IκBα, suggesting that inflammatory responses mediated through other signaling pathways produce synergistic effects on signal transduction in the NF-κB pathway. Taken together, these works highlight the strategies and mechanisms by which cells encode information about multiple ligand combinations and sequences into NF-κB dynamics as a means to interpret complex signaling environments (Fig. 4C).

These studies on the integration of stimulus sequence and combination in NF-κB signaling have revealed key facets of inflammatory processing through the NF-κB network. Cross-tolerance between multiple stimuli has been recognized in the case of IRAK1 and IκBδ, which are activated by one stimulus but act broadly to suppress signaling through many others. Furthermore, testing the effects of prior stimulus history and combinatorial stimuli on signal processing through NF-κB pathway revealed that the NF-κB pathway encodes information about complex environmental dynamics in its oscillatory behavior. The impact of these altered NF-κB oscillations on transcriptional output and cellular response at the single-cell level remains largely unexplored. Furthermore, much of this work has been done with fibroblasts, and investigating the same questions in other immune cell types may reveal alternative strategies of regulation.

## Conclusion

In this review, we highlight studies that show the influence of different stimulus dynamics, sequences, and combinations on the NF-κB response and the subsequent expression of target genes. Many of these studies utilize their findings to reveal the detailed characteristics of the NF-κB network, which are not apparent when investigated with continuous stimulation. Conventional understanding of immune regulatory networks relies on endpoint measurements or results from continuous stimulation, which do not capture the complex dynamics of the signals and signaling networks in vivo. Relying on findings from endpoint measurements to understand the physiological responses can lead to misleading conclusions. For example, by systematically comparing serum TNF-α levels in sepsis patients from various studies, authors of a recent review concluded that TNF-α levels showed a low correlation with the severity of symptoms, although sustained elevated TNF-α levels corresponded to mortality in general^111^. Other studies have shown that a single time point measurement of high levels of cytokines showed a low correlation with severity and mortality in autoimmune diseases^112,113^. Emphasizing the importance of dynamic measurements, an exemplary study showed that cytokine levels actively fluctuate in chronic fatigue syndrome over the course of the illness and suggested that any one-time measurement of cytokines cannot indicate the duration or severity of the disease^114^. Another recent study, which quantified IL-6 levels in patients with septic shock, showed that increasing or decreasing levels of IL-6 were far superior predictor of sepsis mortality than absolute levels of IL-6^115^. These findings suggest the significance of quantifying cytokine level dynamics over the course of diseases and evaluating the effect of dynamics on immune responses.

Other studies focusing on different signaling pathways similarly underscored the significance of investigating network responses to stimulus dynamics. For instance, when the concentration of epidermal growth factor (EGF) was gradually increased in human mammary epithelial cells, extracellular signal-regulated kinase (ERK) activation remained minimal, even as the EGF levels surpassed the threshold for robust activation, which the authors reported stems from the depletion of EGF receptors^116^. In a separate study, a similar effect governed the response of the MAPK/ERK pathway to osmotic stress induced by NaCl^117^. Slow increases in NaCl level in a yeast model prompted the signaling network to gradually adapt to changes in stress levels. In their study, this adaptation ultimately led to the failure to respond adequately to a critical stress dose, resulting in reduced survival rates post-stress. Moreover, another investigation revealed that pulsatile growth factor stimulation influenced cell fate determination independent of the type of growth factor^118^, which is reminiscent of the alterations in NF-κB dynamics during pulsatile stimulation. In a broader theoretical context, a computational study highlighted how testing with diverse stimulation dynamics yielded more precise predictions of signaling network topology and parameters^119^. These examples collectively suggest the widespread occurrence of stimulus-driven changes in pathway behavior across diverse signaling pathways. They also suggest that future research in signaling networks can substantially benefit from investigating the impacts of various stimulus dynamics.

The change in NF-κB response due to dynamic stimulus significantly affects the expression of response genes and can shape the course of an immune response. Although how different NF-κB dynamics precisely modulate inflammatory gene expression is still an active area of research, recent studies have begun to shed light on this question. Different NF-κB translocation dynamics at the single-cell level were correlated with the expression profiles of proinflammatory genes^120^. More specifically, a subpopulation of proinflammatory genes was not upregulated in cells that exhibited a shorter duration of initial NF-κB oscillation. Furthermore, decoding of NF-κB activation duration through chromatin remodeling significantly affected gene expression^16^. In macrophages, stimuli that induced oscillations in NF-κB dynamics have been found to trigger unique and lasting epigenetic changes^14^. Together, these results show how different properties of NF-κB dynamics, such as amplitude, duration, and oscillation, can alter proinflammatory gene expression and the epigenetic state of cell.

Thus, it is important to consider ligand kinetics when studying the immune network or when developing therapies for immune diseases. However, many of the previous studies introduced in this review were also limited by the use of permanent or cancer cell lines, whose responses may differ from those of more physiologically relevant cells. Newly established mouse models with endogenously tagged NF-κB subunits enable a shift in the future focus to primary cell cultures and even intravital imaging of in vivo cytokine responses^11,121,122^. Future investigations utilizing primary cells and mouse models of disease can enhance our understanding of the cellular processing of stimulus dynamics and provide new frameworks for signal processing during infection and under inflammatory conditions.
